# Fasciculation and elongation zeta‐1 protein (FEZ1) interacts with the retinoic acid receptor and participates in transcriptional regulation of the *Hoxb4* gene

**DOI:** 10.1002/2211-5463.12338

**Published:** 2017-12-09

**Authors:** Mariana Bertini Teixeira, Ana Carolina M. Figueira, Ariane S. Furlan, Bruno Aquino, Marcos R. Alborghetti, Adriana F. Paes Leme, Li‐Na Wei, Jörg Kobarg

**Affiliations:** ^1^ Department of Biochemistry and Tissue Biology Institute of Biology University of Campinas Brazil; ^2^ Spectroscopy and Calorimetry Laboratory Brazilian Biosciences National Laboratory Center for Research in Energy and Materials Campinas SP Brazil; ^3^ Department of Biochemistry Universität zu Köln Germany; ^4^ Structural Genomics Consortium University of Campinas Brazil; ^5^ Department of Pharmaceutical Sciences School of Health Sciences University of Brasilia Brazil; ^6^ Mass Spectrometry Laboratory Brazilian Biosciences National Laboratory Center for Research in Energy and Materials Campinas SP Brazil; ^7^ Pharmacology Department University of Minnesota Medical School Minneapolis MN USA; ^8^ Faculty of Pharmaceutical Sciences University of Campinas Brazil

**Keywords:** FEZ, gene regulation, nuclear function, RAR, retinoic acid, transcription

## Abstract

Fasciculation and elongation zeta‐1 (FEZ1) protein is involved in axon outgrowth and is highly expressed in the brain. It has multiple interaction partners, with functions varying from the regulation of neuronal development and intracellular transport mechanisms to transcription regulation. One of its interactors is retinoic acid receptor (RAR), which is activated by retinoic acid and controls many target genes and physiological process. Based on previous evidence suggesting a possible nuclear role for FEZ1, we wanted to deepen our understanding of this function by addressing the FEZ1–RAR interaction. We performed *in vitro* binding experiments and assessed the interface of interaction between both proteins. We found that FEZ1–RAR interacted with a similar magnitude as RAR to its responsive element DR5 and that the interaction occurred in the coiled‐coil region of FEZ1 and in the ligand‐binding domain of RAR. Furthermore, cellular experiments were performed in order to confirm the interaction and screen for induced target genes from an 86‐gene panel. The analysis of gene expression showed that only in the presence of retinoic acid did FEZ1 induce *hoxb4* gene expression. This finding is consistent with data from the literature showing the *hoxb4* gene functionally involved in development and acute myeloid leukemia, as is FEZ1.

**Abbreviations**
AT: all‐trans retinoic acid
DBD: DNA binding domain
DR5: direct repetition with 5 spacer nucleotides
DSS: disuccinimidyl suberate
FITC: fluorescein isothiocyanate
LBD: ligand‐binding domain
RA: all‐trans retinoic acid
RARE: retinoic acid‐responsive element

The fasciculation and elongation zeta/zygin 1 (FEZ1) protein was first identified as a mammalian orthologue of the *Caenorhabditis elegans* UNC‐76 protein and functionally related to axonal outgrowth, especially bundling and elongation 1. Experiments in rat and mouse showed high expression of FEZ1 mRNA in the adult brain, and during development, there was peak of expression with subsequent decrease as development continued 2, 3. Moreover, the knockout mice presented hyperactivity and altered response to psychostimulants 4.

Structurally, FEZ1 contains 392 amino acids and is a natively unfolded protein which dimerizes through a disulfide bond in its N‐terminal domain 5, 6. The highly conserved C‐terminal presents coiled‐coil regions which are the main sites of protein–protein interactions 7. FEZ1 interactors’ partners are related to several functions like regulation of neuronal development, intracellular transport mechanisms, and transcription regulation 7, 8. Among the transcriptional regulators identified as FEZ1 interactors were proteins such as DRAP1 (NC2α), BAF60a, SAP30L, zinc finger 251, RARA, and SCOCO (BERT, UNC‐69) 7, 8.

The human SCOCO (short coiled‐coil protein) is the orthologue of UNC‐69 in *C. elegans* and BERT in *Gallus gallus*. It has been reported that UNC‐69 is widely expressed in the nervous system and plays a role in axon elongation and fasciculation, cooperating with UNC‐76 (FEZ1) 9. Other studies associated the protein BERT as a regulator of the transcriptional complex of the gene *sox2* and the development of the neural plate in chicken embryos 10. Alborghetti and colleagues (2013) further structurally characterized the complex FEZ1–SCOCO 11.

Retinoic acid receptor (RAR) usually forms heterodimer with retinoic X receptor (RXR) and, upon ligand binding, triggers transcription by recruiting coactivators and the recognition of specific RAREs (retinoic acid‐responsive elements) in the gene promoter region 12. The ligand all‐trans retinoic acid (here referred as RA and AT) is the active metabolite of vitamin A and works as a pleiotropic agent that regulates many target genes and processes such as inhibition of cell proliferation, differentiation, apoptosis, shaping of the embryo, and organogenesis 13.

Taken all these information together and also considering our previous results showing FEZ1‐GFP in nuclear fraction 14, we decided to deepen our studies regarding a possible nuclear role of FEZ1 through the interaction with RAR in the presence and absence of ligand RA.

We performed fluorescence anisotropy assays and chemical cross‐linking between FEZ1 and RAR followed by mass spectrometry analysis in order to, first, understand the dynamics of binding affinity and, then, narrowed down the interface of FEZ1–RAR interaction. Cellular experiments also confirmed this interaction, and we saw both proteins colocalizing in the perinuclear region. Finally, we used human brain cells overexpressing FEZ1 in RTqPCR array to analyze a panel of 86 genes related to retinoic acid signaling. We found that the *hoxb4* gene was highly induced in the presence of FEZ1 and RA. Knockdown of *fez1* validated its role as *hoxb4* inducer.

## Methods

### Protein expression and purification

Fasciculation and elongation zeta‐1 (1‐392) protein fused to an N‐terminal 6xHis‐tag was expressed and purified as previously described 5. Affinity chromatography was followed by size exclusion chromatography with a HiLoad Superdex 260 16/60 column in elution buffer A: 137 mm NaCl, 2.7 mm KCl, 10 mm Na_2_HPO_4_, 1.8 mm KH_2_PO_4_, pH 7.4. Aliquots of each eluted fraction obtained were analyzed by SDS/PAGE. Soluble RARΔAB‐pET28a‐His‐tag (lacks N‐terminal; contains DBD and LBD) was purified from 1 L of culture of *Escherichia coli* BL21‐CodonPlus cells that were induced for 16 h to protein expression at 22 °C using 0.5 mm isopropylthio‐β‐D‐galactopyranoside. Cells were harvested by centrifugation at 5000 ***g*** for 15 min, and the cell pellet was resuspended and incubated for 30 min with lysis buffer (20 mm Hepes, 300 mm NaCl, and 5% glycerol, pH 8.0), 100 μm phenylmethylsulfonylfluoride, 2 μm beta‐mercaptoethanol, and protease inhibitor. Lysozyme was added and the lysate incubated for 50 min on ice with occasional shaking. The lysate was sonicated (50 cycles) and then centrifuged at 23 000 ***g*** for 50 min. Affinity chromatography was performed with a peristaltic pump. Running buffer A contained 20 mm Hepes, 300 mm NaCl, 5% glycerol, 5 mm imidazole, pH 8.0, and 2 μm beta‐mercaptoethanol. For the elution buffer B, 300 mm imidazole was added to the running buffer A. Aliquots of each eluted fraction obtained were analyzed by SDS/PAGE, and peak fractions were submitted to molecular exclusion chromatography with a HiLoad Superdex 260 16/60 column. The same was performed for RXRΔAB 15. Purified fractions of proteins were used for anisotropy assays and cross‐linking/MS analysis.

### Fluorescence anisotropy

Assay was performed using an ISS‐PC1 spectrofluorometer (ISS, Champaign, IL), assembled in ‘L’ geometry. Excitation was set to 480 nm, and emission at 520 nm was recorded through an orange short‐wave cutoff filter OG515 (cutoff 50% at 515 nm). Anisotropy values were calculated as described in previous studies 16 using origin software (version 8.0; OriginLab Corp), which applies the Levenberg–Marquardt algorithm for fitting curves to nonlinear equations, to determine the affinity constant (Kd) and Hill coefficient values. All fluorescence experiments were performed in triplicate.

### Titrimetric assay of the FEZ1:RAR interaction

To verify FEZ1:RAR interaction, growing concentrations of FEZ1 (to achieve 1 nm, 5 nm nm, 10 nm, 20 nm, 50 nm, 100 nm, 200 nm, 500 nm, 1000 nm, 2000 nm, and 5000 nm final concentration) were titrated into 50 nm of FITC (fluorescein isothiocyanate)‐labeled RAR, in buffer containing 20 mm Tris pH 8.0, 50 mm NaCl, and 2 mm EDTA. The fluorescence anisotropy measurements were taken at 10 °C.

### Titrimetric assay of the FEZ1:RAR‐DNA interaction

To verify whether the FEZ1:RAR complex is capable to bind RAR‐responsive element DR5 (5′ CGGGTTCACCGAAAGTTCACTCG 3′), increasing amounts of FEZ1:RAR (to achieve 1 nm, 5 nm, 10 nm, 20 nm, 50 nm, 100 nm, 200 nm, 500 nm, 1000 nm, 2000 nm, and 5000 nm final concentration) were titrated into 100 nm of FITC‐DR5, in buffer containing 20 mm Tris pH 8.0, 50 mm NaCl, 2 mm EDTA, and 2 mm MgCl_2_. The fluorescence anisotropy measurements were taken at 10 °C, and the measurements were made both in the presence and in the absence of all‐trans retinoic acid. As controls of this interaction, we performed a series of titrations: (i) RAR into FITC‐DR5, in the presence and absence of all‐trans retinoic acid, and (ii) titration of FEZ1 into FITC‐DR5. All of these were monitored by fluorescence anisotropy, at 10°C, and in each point, final concentration of titrated protein was 1 nm, 5 nm, 10 nm, 20 nm, 50 nm, 100 nm, 200 nM, 500 nm, 1000 nm, 2000 nm, and 5000 nm.

### Titrimetric assay of the FEZ1:RXR‐RAR‐DNA interaction

To verify whether FEZ1 is able to bind RAR:RXR complex in RAR‐responsive element DR5, the complex FEZ1:RAR:RXR (1 nm, 5 nm, 10 nm, 20 nm, 50 nm, 100 nm, 200 nm, 500 nm, 1000 nm, 2000 nm, and 5000 nm final concentration) was titrated into 100 nm of FITC‐DR5, in buffer containing 20 mm Tris pH 8.0, 50 mm NaCl, 2 mm EDTA, and 2 mm MgCl_2_. The fluorescence anisotropy measurements were taken at 10 °C, and the measurements were taken both in the presence and in the absence of all‐trans retinoic acid. As controls of this interaction, we performed a series of titrations: (a) RAR:RXR into FITC‐DR5, in the presence and absence of all‐trans retinoic acid, and (b) FEZ1 into FITC‐DR5. All of these were monitored by fluorescence anisotropy, at 10 °C, and in each point, final concentration of titrated protein was 1 nm, 5 nM, 10 nm, 20 nm, 50 nm, 100 nm, 200 nm, 500 nm, 1000 nm, 2000 nm, and 5000 nm.

### Chemical cross‐linking between 6xHis‐FEZ1 and 6xHis‐RARΔAB followed by mass spectrometry

Purified proteins were incubated (7 μm each) with 1.8 mm disuccinimidyl suberate (DSS; spacer arm length: 11.4 Å, Sigma Aldrich) for 2 h at room temperature, followed by quenching with Laemmli sample buffer. DSS‐cross‐linked complexes were identified as shifted bands in 10% SDS/PAGE. The protein complexes of each band were digested with trypsin. The samples were dried in a vacuum concentrator and reconstituted in 100 μL of 0.1% formic acid. 4.5 μL of the resulting peptide mixture was analyzed in LTQ Orbitrap Velos. Instrument parameters and software analysis were performed as previously described 11, 17. The experiment was performed in duplicate.

### Cell culture and transfection

COS, HeLa, and U87 cells were maintained in Dulbecco's modified Eagle medium (DMEM) supplemented with 10% fetal bovine serum (FBS) and kept at 37 °C and 5% CO_2_ atmosphere. Transient transfection in COS and U87 was performed with Lipofectamine 2000 and 3000 (Thermo Fisher Scientific), respectively, according to the manufacturer's procedures. When prepared to be treated with retinoic acid, serum was changed to 10% charcoal dextran‐coated (DCC).

### Co‐immunoprecipitation

After 48 h of transfection with pCDNAØ (control plasmid), pCDNAFLAG‐FEZ1, and GFP‐RAR, COS cells were harvested and lysed with co‐immunoprecipitation buffer (50 mm Tris pH 8.0, 150 mm NaCl, 0.2% NP‐40, 10% glycerol, 1 mm EDTA, and protease inhibitor), followed by sonication and centrifugation. 260 μg of each lysate was incubated with ANTI‐FLAG M2 Affinity Gel (Sigma Aldrich), and subsequent steps were performed according to the manufacturer's procedures. Elution was performed by the addition of SDS/PAGE sample buffer. Samples were subjected to 10% SDS/PAGE followed by western blot. Membranes were incubated with rabbit anti‐FEZ1 (1 : 500, Novus Biologicals, NBP1‐86256) or rabbit anti‐GFP (1 : 1000, Santa Cruz, sc‐8334) primary antibodies, and goat anti‐rabbit HRP (1 : 2000, Santa Cruz, sc‐2004) secondary antibody.

### Immunofluorescence

HeLa cells plated on a 24‐well dish were fixed and permeabilized with 3.7% formaldehyde and 0.2% Triton X‐100. Cells were incubated in buffer containing 7.5 mg·mL^−1^ glycine and then blocked with 3% bovine serum albumin and 0.1% Triton X‐100 for 30 min at room temperature. The cells were then incubated with anti‐FEZ1 (1 : 100, Atlas, HPA038490) and anti‐RAR (1 : 100, Abcam, ab28767) primary antibodies for 1 h at room temperature and then with chicken anti‐rabbit 488 (1 : 250, Thermo Fisher, A21441) and donkey anti‐goat 546 (1 : 250, Thermo Fisher, A11056) secondary antibodies for 40 min at room temperature. Nuclei were stained with 1 : 10 000 Hoechst 33342. Slides were examined with Leica confocal microscope.

### FEZ1‐knockdown cell line

FEZ1 shRNA lentiviral particles were purchased from the Genomics Center of the University of Minnesota plus one shGFP control. Three shFEZ1 constructs were tested in U87 cells, and clone selection was performed using 1.75 μg·mL^−1^ puromycin. Efficiency of silencing was assessed using RTqPCR with specific primers for FEZ1 (sense: GACCCTGAGGAAGAAGAGGA; antisense: CAGCCCTTCATAGGACCAGT).

### Analysis of gene expression

After 48 h of transfection with pCDNAØ and pCDNAFLAG‐FEZ1, U87 cells were treated with 300 nm all‐trans retinoic acid for 24 h. RNA was extracted using TRIzol (Invitrogen). 2 μg RNA was reverse‐transcribed using MultiScribe Reverse Transcriptase (Thermo Fisher). RTqPCR was performed with Stratagene Mx3005P; reactions contained 2x Maxima SYBR Green qPCR Master Mix (Thermo Fisher). In order to assess transfection efficiency and all‐trans retinoic acid activity, cDNA and specific primers for *fez1* and *rar beta* were used and the results normalized with *beta actin*. Furthermore, RT Profiler PCR Array (QIAGEN) with 86 genes plus 5 controls related to human retinoic acid signaling (330231) was used for samples overexpressing FEZ1 and then confirmed with FEZ1 knockdown. Equipment settings, sample handling, and analyses were performed according to the manufacturer's procedures.

## Results

### Dynamics of protein–protein interaction and DNA–protein interaction by fluorescence anisotropy

**Figure 1:**
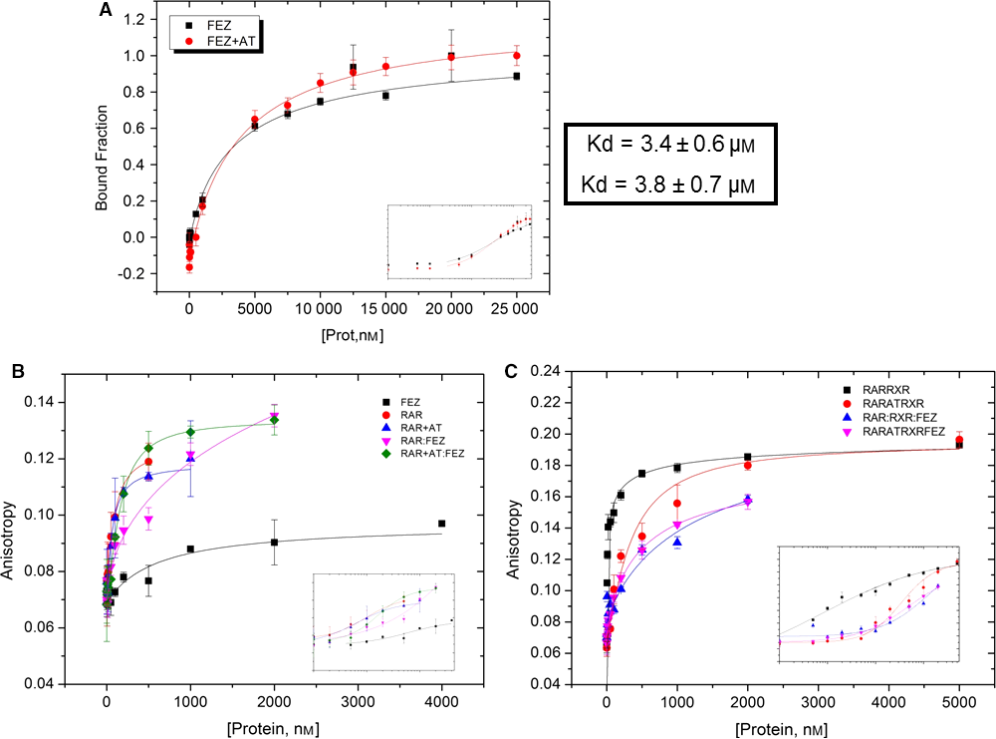
Relative binding affinity of FEZ1‐RAR. (A) Protein–protein interaction: FEZ1 titrated on RAR‐FITC in the presence and absence of all‐trans retinoic acid (AT). (B) and (C) Anisotropy curves from protein (FEZ1, RAR, RAR:RXR) titrated on DR5‐FITC probe in the absence and presence of AT.

In order to gather information about binding affinity and interaction dynamics, we performed anisotropy assay with both proteins in the absence and presence of all‐trans retinoic acid (AT) (Fig. 1A). We found that the dissociation constant of the interaction (Kd) in the absence of ligand was 3.4 ± 0.6 μm and in the presence of ligand was 3.8 ± 0.7 μm, meaning that the ligand did not affect this protein–protein interaction.

In addition, we also used the same technique to evaluate the interaction between FEZ1 and RAR to a DNA fragment (fluorophore‐labeled reporter DR5). Both purified RAR and FEZ1 were titrated, in the absence and presence of the ligand all‐trans retinoic acid (AT) (Fig. 1B). Moreover, as it is known that RAR also binds DNA as a heterodimer, the same experiment was performed with the heterodimer RAR–RXR to verify the role of RXR in the complex DR5–RAR–FEZ1 (Fig. 1C).

From the obtained Kd values (Table 1), it is possible to observe that RAR, RAR+AT, and FEZ1:RAR+AT all have comparable Kd when we consider that the values may vary positively or negatively, meaning that the presence of FEZ1 did not significantly disturb the binding of RAR to DR5 when the ligand retinoic acid (AT) was present (FEZ1:RAR+AT). We can see that the presence of FEZ1 alone did not bind DNA and also that FEZ1:RAR without ligand was the one with the weakest binding affinity. Lastly, the Hill coefficient (*n*) was higher in FEZ1:RAR+AT, indicating that the binding of one molecule could facilitate the binding of subsequent molecules to the complex. But again, when we consider the errors, the values could change positively or negatively and therefore they are practically the same for all situations.

**Table 1:** Quantitative analysis of the interaction of RAR with DR5‐FITC in the absence and presence of ligand all‐trans retinoic acid (AT) and in the absence and presence of FEZ1

	K_d_(nm)	Hill *n*
RAR	94 ± 43	1.2 ± 0.3
RAR+AT	64 ± 13	1.1 ± 0.2
FEZ	–	–
FEZ:RAR	385 ± 102	1.0 ± 0.2
FEZ:RAR+AT	162 ± 11	1.4 ± 0.1

The experiment with RAR:RXR showed a similar situation (Table 2). The binding affinity of RAR:RXR to DNA was the highest, as expected. The presence of FEZ1 and ligand (FEZ1:RAR+AT:RXR) was comparable to the situation RAR+AT:RXR. Once more, the presence of FEZ1 without ligand (FEZ1:RAR:RXR) showed the weakest binding affinity. The Hill coefficient showed practically a similar value for all conditions when we consider the positive or negative variations.

**Table 2:** Quantitative analysis of the interaction of RAR:RXR with DR5‐FITC in the absence and presence of ligand all‐trans retinoic (AT) acid and in the absence and presence of FEZ1

	K_d_(nm)	Hill *n*
RAR:RXR	18.5 ± 2.1	0.6 ± 0.1
RAR+AT:RXR	347.1 ± 103	1.2 ± 0.2
FEZ:RAR:RXR	755 ± 131	0.8 ± 0.4
FEZ:RAR+AT:RXR	480.6 ± 180	1.3 ± 0.2
FEZ	–	**–**

Taken together, these data confirmed the binding affinity between RAR and RAR:RXR to its responsive element DR5 and also showed that *in vitro* the presence of ligand all‐trans retinoic acid may cause some structural conformational change that seemed to favor the binding of FEZ1 to RAR and also to the heterodimer RAR:RXR.

### Chemical cross‐linking coupled with mass spectrometry

**Figure 2:**
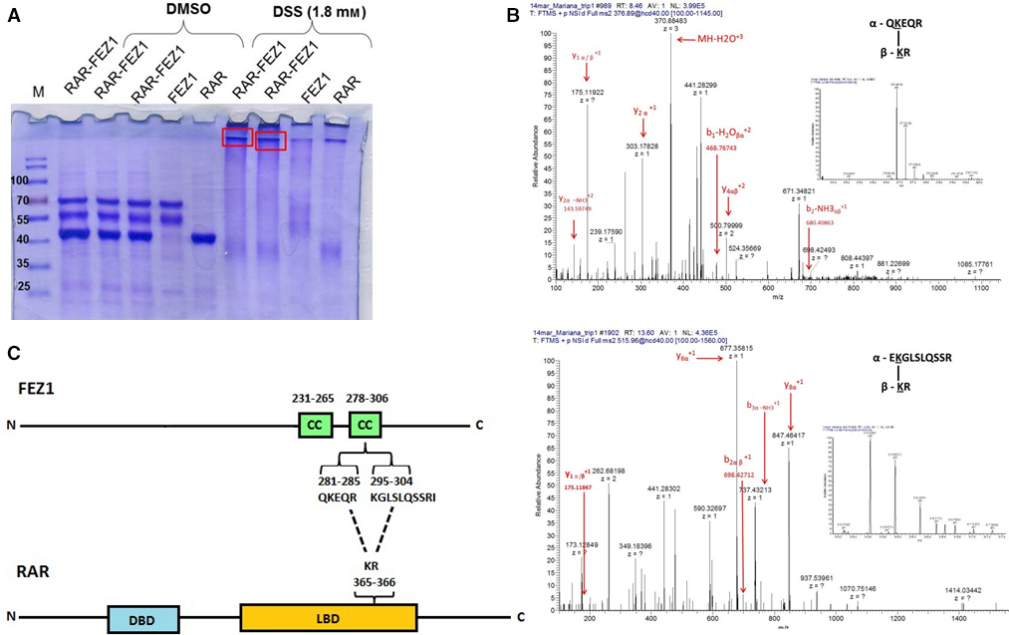
Binding interface of the FEZ1–RAR interaction. (A) Recombinant purified proteins were incubated with DSS and DMSO (control). Bands corresponding to complexes (red rectangles) were excised from polyacrylamide gel, digested with trypsin, and analyzed by LC‐MS/MS. (B) Spectra were manually validated for b and y ion series of the α (peptides of FEZ1) and β (peptides of RAR) chains. (C) Schematic representation of FEZ1 and RAR. FEZ1 presents two coiled‐coils (CC) predicted regions (231‐265 amino acids; 278‐306 amino acids). In RAR, the DBD (DNA binding domain) and LBD (ligand‐binding domain) are represented. Two predicted interaction sites were found for FEZ1, ranging from amino acids 281‐285 (QKEQR) and 295‐304 (KGLSLQSSRI). These two regions are in one of the FEZ1 coiled‐coil. The predicted binding region for RAR is located in its LBD, amino acids 365‐366 (KR). M = molecular mass marker. The indicated molecular masses of the marker protein bands are shown in kDa.

After the initial identification of the interaction between FEZ1 and RAR in yeast two‐hybrid assays 8 and performing the anisotropy fluorescence experiments above, we were interested in mapping the interface of interaction between these two proteins by chemical cross‐linking coupled with mass spectrometry. After incubating both purified proteins with cross‐linker DSS and running SDS/PAGE, bands were cut from the gel (Fig. 2A). Note that the polyacrylamide gel also contains the following controls: RAR‐FEZ1 incubated without DMSO (vehicle) nor DSS, RAR‐FEZ1 with DMSO alone, FEZ1 alone with DMSO, and RAR alone with DMSO. After addition and incubation with DSS, there was clearly a change in the migration pattern of proteins in complex and intense, higher, bands could be seen on the gel, indicating that the complex may have been formed. In order to confirm this and also to verify which amino acids from each protein were responsible for the interaction and complex formation, the samples were digested with trypsin and prepared for LC‐MS/MS analysis (Fig. 2B). Two possible regions of contact in FEZ1 were identified ^281^QKEQR^285^ and ^295^ KGLSLQSSRI^304^ and one in RAR ^365^ KR^366^ (Fig. 2C).

These results were consistent with previous data from the literature and from our group, confirming that the interaction of proteins with FEZ1 occurs in its main protein interaction docking domain, the coiled‐coil regions (predicted coiled‐coils: amino acids 231‐265 and 278‐306). Furthermore, we found that FEZ1 binds to RAR in the ligand‐binding domain (LBD), which is described as the region where other proteins bind to and form complexes that regulate transcription. This finding opened the possibility that FEZ1 could be part of a larger transcription regulatory complex built around RAR that may regulate expression of target genes.

### Characterizing FEZ1 and RAR interaction in mammalian cells

**Figure 3:**
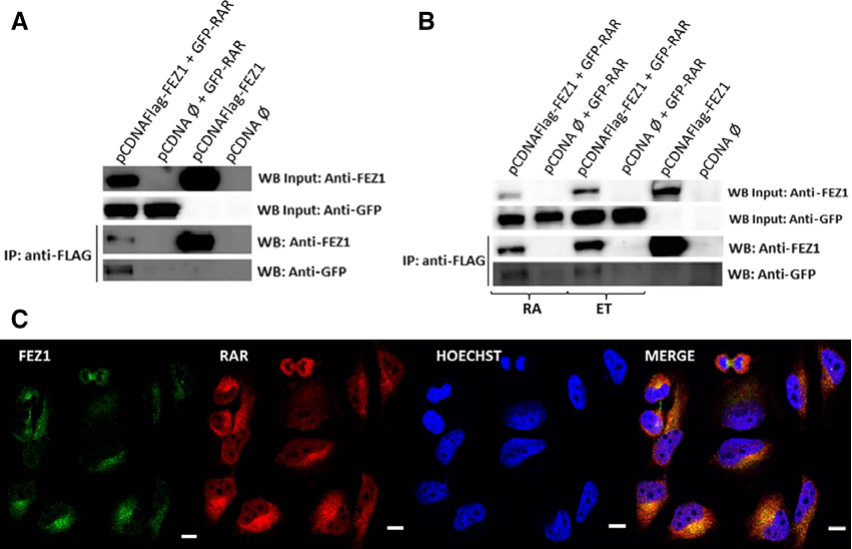
FEZ1 and RAR interaction and localization in cells. (A) Overexpression of FEZ1 and RAR in COS cells and co‐immunoprecipitation using FLAG resin, followed by western blot. (B) Samples were treated with ethanol (ET; control) or all‐trans retinoic acid (RA, dissolved in ethanol) for 24 h and then co‐immunoprecipitated. (C) HeLa cells were analyzed by immunocytochemistry using anti‐FEZ1 (green) and anti‐RAR (red) antibodies and the nuclear dye Hoechst (blue). Colocalization occurred in the perinuclear region (yellow, merge image). White scale bar = 0.2 inches.

Aiming to confirm the interaction between FEZ1 and RAR also in cells, we performed co‐immunoprecipitation (IP) experiments in COS cells. First, we transfected FLAG‐FEZ1 and GFP‐RAR and then incubated the lysate with anti‐FLAG beads. After developing western blot membranes marked with anti‐GFP and anti‐FEZ1 primary antibodies, we confirmed that RAR and FEZ1 were co‐immunoprecipitated (Fig. 3A).

In the next co‐immunoprecipitation, we wanted to understand how this complex would behave in the presence of ligand all‐trans retinoic acid (RA) when compared with control. After transfecting cells, we treated them with 500 nm RA for 24 h. Lysates were again incubated with beads followed by western blot. As depicted in Fig. 3B, we could see that the interaction continued to occur in the presence of ligand.

Next, immunostaining of endogenous RAR and FEZ1 was performed in HeLa cells in order to visualize the location of both proteins using confocal microscopy. As seen in Fig. 3C, FEZ1 appears mainly in the cytoplasm, while RAR can be seen both in nucleus and in cytoplasm. Regarding the colocalization of both proteins, it occurred in the perinuclear region, without any ligand treatment.

### Gene expression profile after overexpression and silencing of FEZ1

Upon confirmation of FEZ1–RAR interaction and the possible role of FEZ1 as a participant in transcription, we wanted to evaluate the target genes that could be induced by overexpression of FEZ1 and then confirm the regulation of the candidate genes by depletion of FEZ1. In order to test a variety of genes that are known to be part of retinoic acid signaling, we used a commercial array that contains 86 genes plus 5 control genes.

**Figure 4:**
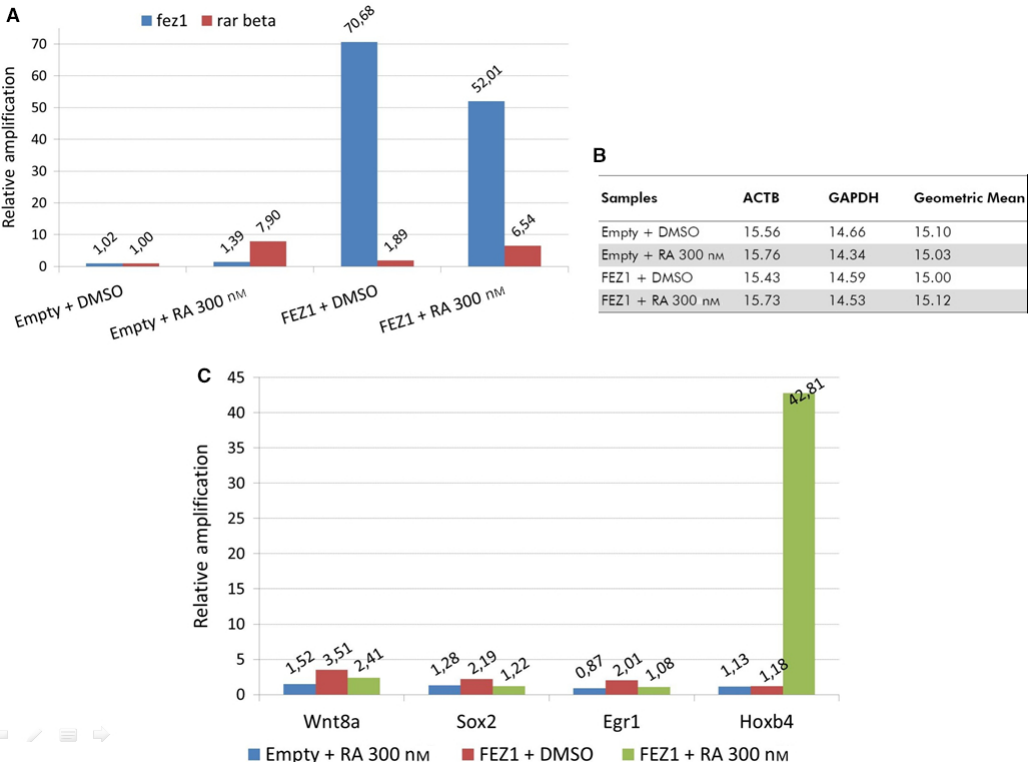
Overexpression of FEZ1 and gene expression analysis. (A) Confirmation of *fez1* overexpression and activation of *rar beta* by retinoic acid (RA). (B) From a panel of five control genes, *beta actin* (ACTB) and *gapdh* were automatically selected for relative quantification, and the results were analyzed by the ΔΔCt method. Group ‘empty + DMSO’ was selected to normalize the samples. (C) Genes that were differentially induced in groups ‘FEZ1 + DMSO’ and ‘FEZ1 + RA 300 nm’. Genes from group ‘Empty + RA 300 nm’ are depicted for comparison and were not activated.

We designed the overexpression experiment with four groups: pCDNA empty plasmid (control) + DMSO (vehicle) treatment, FEZ1‐pCDNA + DMSO, pCDNA empty plasmid + 300 nm RA, and FEZ1‐pCDNA + 300 nm RA (Fig. 4). After extracting the RNA and producing cDNA, the samples from each group were added to the commercial array with SYBR Green and RTqPCR analyses were performed. In addition, due to the described neuronal functions of FEZ1 1, 4, the human cell line U87 derived from brain was used as the model to perform the studies of gene expression.

**Figure 5:**
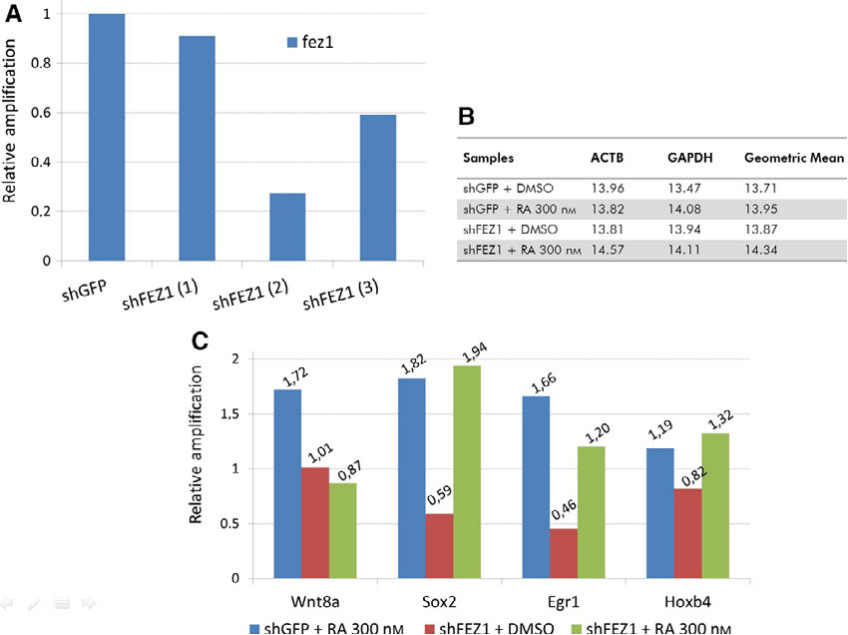
Depletion of FEZ1 and gene expression analysis. (A) Three shFEZ1 (plus shGFP as a control) were tested and shFEZ1(2) was selected to subsequent studies (~ 80% silenced). (B) From a panel of five control genes, *beta actin* (ACTB) and *gapdh* were automatically selected for relative quantification, and the results were analyzed by the ΔΔCt method. Group ‘shGFP + DMSO’ was selected to normalize the samples. (C) Genes are the same from Fig. 4C. The low values, below or close to 1.0, mean that there was no gene activation, confirming the results obtained with the overexpression situation.

In order to confirm that the genes induced when there was overexpression of FEZ1 would not be activated when FEZ1 was depleted, and therefore showing that FEZ1 was indeed responsible for determined gene induction, we generated *fez1*‐knockdown U87 cells using shRNA lentiviral infection. Three FEZ1 shRNA were tested and we also used one GFP shRNA as a control. After analyzing the efficiency of silencing using RTqPCR with *fez1*‐specific primers, we selected the one that provided approximately 80% of depletion (Fig. 5A) and performed the experiment with the commercial array once again. This time the groups were shGFP (control) + DMSO (vehicle), shFEZ1 + DMSO, shGFP + 300 nm RA, and shFEZ1 + 300 nm RA. The statistical analyses were performed by normalization with *actin* and *gapdh* obtained values, and further with the sample group ‘control/empty plasmid + DMSO’.

When FEZ1 was overexpressed, we could see that three genes [wnt8a (3.51‐fold), sox2 (2.19‐fold), and egr1 (2.01‐fold)] were differentially induced in the absence of RA, and 2 genes [hoxb4 (42.66‐fold) and wnt8a (2.40‐fold)] were differentially induced in the presence of RA, when compared to genes from the ‘empty + RA’ condition (Fig. 4C). In Fig. 4A, we confirm the overexpression of *fez1* and the activation of *rar beta* in the conditions where RA was added. In Fig. 4B, the control genes *beta actin* and *gapdh* were automatically selected by the analysis software to be used for normalization.

The three genes for ‘FEZ1 + DMSO’ showed that the fold‐regulation value ranged from 3.5 to 2.2 and here we want to highlight the activation of the gene *sox2* (and the lack of its activation in the FEZ1‐depleted situation—Fig. 5C, red bar—suggesting that FEZ1 was really involved in this gene activation)*,* which is consistent with previous findings 10, that associated *sox2* regulation with BERT, the chicken orthologue of FEZ1 interactor SCOCO.

For the two genes present in ‘FEZ1 + RA’ group, we want to highlight *hoxb4* that was dramatically induced (42.66‐fold, Fig. 4C, green bar). Conversely, the depletion of *fez1* implicated in reduced *hoxb4* expression (Fig. 5C, green bar), suggesting that FEZ1 might really play a role in the transcription of *hoxb4*. The gene *wnt8a* was 2.40‐fold induced (and also appeared induced in ‘FEZ1 + DMSO’, suggesting that its activation is dependent on FEZ1 and independent of ligand).

## Discussion

Fasciculation and elongation zeta‐1 is a protein with multiple interaction partners 7, 8 and has been described mainly as a cytoplasmic protein. After findings of a) FEZ1 interacting with nuclear proteins and more specifically, the retinoic acid receptor 8; b) FEZ1‐GFP present in nuclear fraction of HEK cells 14; and c) BERT, orthologue of permanent FEZ1 interactor SCOCO, as a regulator of *sox2* 10; we decided to investigate the nuclear role of FEZ1 through its interaction with RAR.

We performed *in vitro* studies with recombinant purified proteins and found that protein–protein interaction between FEZ1‐RAR was not disturbed by the presence of all‐trans retinoic acid (RA). Consistently, the co‐immunoprecipitation experiments confirmed that FEZ1 and RAR were co‐immunoprecipitated both in the absence and in the presence of RA.

When we performed titration of purified FEZ1 and RAR on a DR5 fluorescent probe to analyze how the complex DR5–RAR and DR5‐RAR:RXR would behave in the presence of FEZ1, we found that the presence of both FEZ1 and all‐trans retinoic acid (FEZ1:RAR+AT and FEZ1:RAR+AT:RXR) did not disturb the complexes, as the interaction occurred with similar dissociation constants as the ones for RAR and RAR:RXR to its responsive element DR5.

Regarding the cellular location, the immunofluorescence showed that both proteins were in the perinuclear region without any ligand treatment. We look forward to perform more imaging experiments in the future aiming to address FEZ1 translocation, especially because previous and preliminary results from our group (not shown) have reported the presence of FEZ1 in the nucleus upon treatment with RA. Supporting this evidence, it has been reported that FEZ1 possess a nuclear localization signal in its C‐terminal and subcellular fractionation in HEK cells showed the presence of FEZ1 in both cytoplasmic and nuclear fractions 14.

In addition, the chemical cross‐linking followed by mass spectrometry demonstrated that the binding interface involves the C‐terminal coiled‐coil region of FEZ1 and the ligand‐binding domain of RAR (LBD). In strong accordance with this finding, it has been extensively reported that the FEZ1 C‐terminal is the main region of protein–protein interaction, and proteins like kinesin, DISC1, agnoprotein, PKCζ, and others have been shown to bind FEZ1 in this region 18. Moreover, the LBD of RAR is also characterized as the domain where a number of regulatory proteins bind in a dynamic way, depending also on the presence or absence of ligand, which will determine gene transcription activation or repression 19.

Finally, we performed a screen to investigate the target genes that could be activated by FEZ1 as part of a predicted transcription regulatory complex. The gene expression analysis using an array of genes related to retinoic acid signaling and cells derived from brain (the tissue where FEZ1 has high expression levels) showed that *sox2* gene was induced when FEZ1 was overexpressed and this was confirmed when FEZ1 was depleted and there was no *sox2* activation, supporting the hypothesis that the presence of FEZ1 could be part of *sox2* activation. This finding is very consistent with previous results 10 from the orthologue of human SCOCO, BERT (*Gallus gallus* short coiled‐coil protein), which was reported to participate in regulating the expression of gene *sox2* in the neural plate development, and SCOCO is an important, permanent interaction partner of FEZ1 11.

Most consistently, we also found that the gene *hoxb4* was dramatically induced upon FEZ1 overexpression and treatment with RA. This result was further confirmed when cells were silenced for FEZ1 and treated with RA and there was no *hoxb4* activation, showing that FEZ1 might indeed play a role in *hoxb4* activation. Further studies like ChIP assay could give us more details about the participation of FEZ1 together with RAR in the transcription complex that regulates the h*oxb4* gene.

*Hoxb4* is known to be related to development 20 and experiments from our group showed *fez1* gene expression by *in situ* hybridization in chicken embryos during neurulation and somitogenesis (not shown). Also, as already mentioned, there is a peak of FEZ1 mRNA expression during development in rat and mouse 2, 3. It would be of great interest to further study how FEZ1 correlates with the genes reported here, such as *sox2*,* hoxb4*, and *wnt8a*, in an embryonic developing model.

More surprisingly, HOXB4 was reported to correlate with acute myeloid leukemia 21, which is very consistent with our findings regarding FEZ1 expression in this disease (not published). The interesting link between FEZ1 and HOXB4 differential expression in acute myeloid leukemia could lie in the discovery that FEZ1 overexpression caused the phenotype of multilobulated nuclei (also known as flower‐like nuclei) in mammalian cell line 14. This nuclear phenotype has already been reported to be a marker of myeloid leukemia of M4/5 subtype 22, 23.

In conclusion, our study was the first to characterize, through different techniques, the interaction between FEZ1 and RAR, a nuclear receptor. We are also reporting the first evidence of FEZ1 playing a nuclear role by participating in the regulation of genes, especially the *hoxb4* gene, which presented the highest fold induction in the presence of both FEZ1 and RA. We propose that FEZ1 binds RAR and participates in a protein complex that can activate *hoxb4* gene. This is the mechanism by which retinoic acid binds to RAR and there is a recruitment of proteins that participate in the transcription of genes. We hope that this work will lead to new studies regarding the nuclear role of FEZ1.

## Author contributions

MBT, ASF, BA, MRA, and JK conceived and designed the project; MBT and ASF acquired the data; MBT, BA, ACMF, AFPL, LNW, and JK analyzed and interpreted the data; MBT and JK wrote the manuscript. All authors read, approved, and improved the final version of the manuscript.

## References

[1] Bloom L and Horvitz HR (1997) The *Caenorhabditis elegans* gene unc‐76 and its human homologs define a new gene family involved in axonal outgrowth and fasciculation. Proc Natl Acad Sci 94, 3414–3419. 9096408 10.1073/pnas.94.7.3414 PMC20384

[2] Honda A , Miyoshi K , Baba K , Taniguchi M , Koyama Y , Kuroda S , Katayama T and Tohyama M (2004) Expression of fasciculation and elongation protein zeta‐1 (FEZ1) in the developing rat brain. Mol Brain Res 122, 89–92. 14992819 10.1016/j.molbrainres.2003.11.020

[3] Fujita T , Ikuta J , Hamada J , Okajima T , Tatematsu K , Tanizawa K and Kuroda S (2004) Identification of a tissue‐non‐specific homologue of axonal fasciculation and elongation protein zeta‐1. Biochem Biophys Res Commun 313, 738–744. 14697253 10.1016/j.bbrc.2003.12.006

[4] Sakae N , Yamasaki N , Kitaichi K , Fukuda T , Yamada M , Yoshikawa H , Hiranita T , Tatsumi Y , Kira J , Yamamoto T *et al* (2008) Mice lacking the schizophrenia‐associated protein FEZ1 manifest hyperactivity and enhanced responsiveness to psychostimulants. Hum Mol Genet 17, 3191–3203. 18647754 10.1093/hmg/ddn215

[5] Lanza DC , Silva JC , Assmann EM , Quaresma AJ , Bressan GC , Torriani IL and Kobarg J (2009) Human FEZ1 has characteristics of a natively unfolded protein and dimerizes in solution. Proteins 74, 104–121. 18615714 10.1002/prot.22135

[6] Alborghetti MR , Furlan AS , Silva JC , Paes Leme AF , Torriani ICL and Kobarg J (2010) Human FEZ1 protein forms a disulfide bond mediated dimer: implications for cargo transport. J Proteome Res 9, 4595–4603. 20812761 10.1021/pr100314q

[7] Assmann EM , Alborghetti MR , Camargo ME and Kobarg J (2006) FEZ1 dimerization and interaction with transcription regulatory proteins involves its coiled‐coil region. J Biol Chem 281, 9869–9881. 16484223 10.1074/jbc.M513280200

[8] Alborghetti MR , Furlan AS and Kobarg J (2011) FEZ2 has acquired additional protein interaction partners relative to FEZ1: functional and evolutionary implications. PLoS ONE 6, e17426. 21408165 10.1371/journal.pone.0017426 PMC3050892

[9] Su CW , Tharin S , Jin Y , Wightman B , Spector M , Meili D , Tsung N , Rhiner C , Bourikas D , Stoeckli E *et al* (2006) The short coiled‐coil domain‐containing protein UNC‐69 cooperates with UNC‐76 to regulate axonal outgrowth and normal presynaptic organization in *Caenorhabditis elegans* . J Biol 5, 9. 16725058 10.1186/jbiol39 PMC1561584

[10] Papanayotou C , Mey A , Birot AM , Saka Y and Boast S (2008) A mechanism regulating the onset of Sox2 expression in the embryonic neural plate. PLoS Biol 6, 110–123. 10.1371/journal.pbio.0060002 PMC2174969 18184035

[11] Alborghetti MR , Furlan AS , da Silva JC , Sforça ML , Honorato RV , Granato DC , dos Santos Migueleti DL , Neves JL , de Oliveira PS , Paes‐Leme AF *et al* (2013) Structural analysis of intermolecular interactions in the kinesin adaptor complex fasciculation and elongation protein zeta 1/short coiled‐coil protein (FEZ1‐SCOCO). PLoS ONE 8, e76602. 24116125 10.1371/journal.pone.0076602 PMC3792052

[12] Larange A and Cheroutre H (2016) Retinoic acid and retinoic acid receptors as pleiotropic modulators of the immune system. Annu Rev Immunol 34, 369–394. 27168242 10.1146/annurev-immunol-041015-055427

[13] Schug TT , Berry DC , Shaw NS , Travis SN and Noy N (2007) Opposing effects of retinoic acid on cell growth result from alternate activation of two different nuclear receptors. Cell 129, 723–733. 17512406 10.1016/j.cell.2007.02.050 PMC1948722

[14] Lanza DC , Trindade DM , Assmann EM and Kobarg J (2008) Over‐expression of GFP‐FEZ1 causes generation of multi‐lobulated nuclei mediated by microtubules in HEK293 cells. Exp Cell Res 314, 2028–2039. 18439996 10.1016/j.yexcr.2008.02.012

[15] Fattori J , Campos JL , Doratioto TR , Assis LM , Vitorino MT , Polikarpov I , Xavier‐Neto J and Figueira ACM (2015) RXR agonist modulates TR: corepressor dissociation upon 9‐cis retinoic acid treatment. Mol Endocrinol 29, 258–273. 25541638 10.1210/me.2014-1251 PMC5414759

[16] Figueira ACM , Lima LMTR , Lima LHF , Ranzani AT , Mule GS and Polikarpov I (2010) Recognition by the thyroid hormone receptor of canonical DNA response elements. Biochemistry 49, 893–904. 20025240 10.1021/bi901282s

[17] Aragão AZB , Nogueira MLC , Granato DC , Simabuco FM , Honorato RV , Hoffman Z , Yokoo S , Laurindo FRM , Squina FM , Zeri ACM *et al* (2012) Identification of Novel Interaction between ADAM17 (a disintegrin and metalloprotease 17) and Thioredoxin‐1. J Biol Chem 287, 43071–43082. 23105116 10.1074/jbc.M112.364513 PMC3522302

[18] Maturana AD , Fujita T and Kuroda S (2010) Functions of fasciculation and elongation protein Zeta‐1 (FEZ1) in the brain. Sci World J 10, 1646–1654. 10.1100/tsw.2010.151 PMC5763902 20730382

[19] Perissi V and Rosenfeld MG (2005) Controlling nuclear receptors: the circular logic of cofactor cycles. Nat Rev Mol Cell Biol 6, 542–554. 15957004 10.1038/nrm1680

[20] Garcia‐Fernàndez J (2005) The genesis and evolution of homeobox gene clusters. Nat Rev Genet 6, 881–892. 16341069 10.1038/nrg1723

[21] Umeda S , Yamamoto K , Murayama T , Hidaka M , Kurata M , Ohshima T , Suzuki S , Sugawara E , Kawano F and Kitagawa M (2012) Prognostic significance of HOXB4 in *de novo* acute myeloid leukemia. Hematology 17, 125–131. 22664110 10.1179/102453312X13376952196250

[22] Shimoyama M , Kagami Y , Shimotohno K , Miwa M , Minato K , Tobinai K , Suemasu K and Sugimura T (1986) Adult T‐cell leukemia/lymphoma not associated with human T‐cell leukemia virus type I. Proc Natl Acad Sci USA 83, 4524–4528. 3012571 10.1073/pnas.83.12.4524 PMC323766

[23] Graham RL , Burch M and Krause JR (2014) Adult T‐cell leukemia/lymphoma. Proc (Bayl Univ Med Cent) 27, 235–238. 24982574 10.1080/08998280.2014.11929123 PMC4059578

